# Spatial Localization of Sources in the Rat Subthalamic Motor Region Using an Inverse Current Source Density Method

**DOI:** 10.3389/fncir.2016.00087

**Published:** 2016-11-03

**Authors:** Kees J. van Dijk, Marcus L. F. Janssen, Daphne G. M. Zwartjes, Yasin Temel, Veerle Visser-Vandewalle, Peter H. Veltink, Abdelhamid Benazzouz, Tjitske Heida

**Affiliations:** ^1^Biomedical Signals and Systems Group, MIRA institute for Biomedical Engineering and Technical Medicine, University of TwenteEnschede, Netherlands; ^2^Department of Neuroscience, School for Mental Health and Neuroscience, Maastricht UniversityMaastricht, Netherlands; ^3^Department of Neurology, Maastricht University Medical CenterMaastricht, Netherlands; ^4^University de Bordeaux, Institut des Maladies Neurodégénératives, Centre National de la Recherche Scientifique UMR 5293Bordeaux, France; ^5^Department of Neurosurgery, Maastricht University Medical CenterMaastricht, Netherlands; ^6^Department of Stereotactic and Functional Neurosurgery, University of CologneCologne, Germany

**Keywords:** inverse current source density analysis, local field potentials, action potentials, cortical stimulation, subthalamic nucleus, rodents

## Abstract

**Objective:** In this study we introduce the use of the current source density (CSD) method as a way to visualize the spatial organization of evoked responses in the rat subthalamic nucleus (STN) at fixed time stamps resulting from motor cortex stimulation. This method offers opportunities to visualize neuronal input and study the relation between the synaptic input and the neural output of neural populations.

**Approach:** Motor cortex evoked local field potentials and unit activity were measured in the subthalamic region, with a 3D measurement grid consisting of 320 measurement points and high spatial resolution. This allowed us to visualize the evoked synaptic input by estimating the current source density (CSD) from the measured local field potentials, using the inverse CSD method. At the same time, the neuronal output of the cells within the grid is assessed by calculating post stimulus time histograms.

**Main results:** The CSD method resulted in clear and distinguishable sources and sinks of the neuronal input activity in the STN after motor cortex stimulation. We showed that the center of the synaptic input of the STN from the motor cortex is located dorsal to the input from globus pallidus.

**Significance:** For the first time we have performed CSD analysis on motor cortex stimulation evoked LFP responses in the rat STN as a proof of principle. Our results suggest that the CSD method can be used to gain new insights into the spatial extent of synaptic pathways in brain structures.

## Introduction

In the last decades, technology for the recording of neuronal activity has advanced rapidly. Probes and microelectrode arrays have become available, which allow electrophysiological recordings with high temporal and spatial resolution (Buzsáki, 2004; Kipke et al., 2008; Du et al., 2011). In general, the recordings of neuronal activity can be divided into two components: The high frequency part of the potentials measured provide information about the spiking activity of neurons nearby, while the low-frequency part (the local field potential; LFP) contains information about how the dendrites process synaptic inputs (Buzsáki et al., 2012). The recorded potentials are dominated by a weighted sum of contributions from transmembrane currents through the membranes of the neurons nearby the electrode contacts (Buzsáki et al., 2012). Unfortunately, the large number of contributing sources makes the interpretation of the recordings complicated. Therefore, careful mathematical modeling and analysis are needed to take full advantage of the opportunities that such measurements offer in understanding the signal processing in neurons and neural circuits (Einevoll et al., 2013). The development of methods for such modeling and signal analysis becomes even more pertinent with the on-going technological advancement. For example in the field of deep brain stimulation, novel stimulation lead designs (Martens et al., 2011; van Dijk et al., 2015) allow LFP recordings on multiple locations within the region of interest to identify the stimulation target (Bour et al., 2015).

When grouped synaptic activity is sufficiently synchronized, it is often evident at the level of the LFP (Hubbard et al., 1969; Mitzdorf, 1985). By stimulating pre-synaptic neuronal populations, it is possible to evoke synchronized synaptic input in post-synaptic neuronal populations. The synaptic activation will cause an inflow of ions at the dendrites. For example, an inhibitory synaptic input using gamma-aminobutyric acid (GABA) as a neurotransmitter will cause an inflow of negatively charged Chloride (Cl^−^) ions at the dendrites. An excitatory synaptic input using glutamate as a neurotransmitter will cause an inflow of positively charged Sodium (Na^+^) and Potassium (K^+^) ions at the dendrites (Purves, 2008). The ionic flow in and out of the extracellular medium caused by synaptic input can be described by the current source density (CSD) (Einevoll et al., 2013). LFP recording with high spatial resolution microelectrode arrays allows us to estimate the CSD from the LFPs (Freeman and Nicholson, 1975; Mitzdorf, 1985; Pettersen et al., 2006; Leski et al., 2007). With the CSD one can study the occurrence, spatial distribution and extent of the current sources and sinks of the synaptic input more locally. This offers opportunities to visualize neuronal input and study the relation between the synaptic input and the neural output, i.e., unit activity, of neural populations.

**Figure 1:**
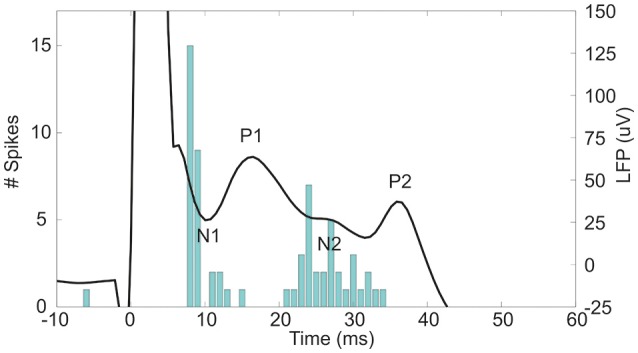
**The evoked LFP and peristimulus time histogram (PSTH) of the unit activity in the STN after 600 μA MCS**. At 0 ms the stimulus is given. The negative deflections of the LFP, N1, and N2, are accompanied by an increased spiking rate as seen in the PSTH. In contrast, the positive deflections, P1 and P2, occurred concurrently with a decreased spiking rate in the PSTH.

The subthalamic nucleus (STN) is an important relay in the basal ganglia network as it is one of the main entry ports at which cortical input enters the basal ganglia and modulates the basal ganglia output structures (Parent and Hazrati, 1995; Nambu et al., 2002). Cortical signals are conveyed to the STN by the monosynaptic cortico-subthalamic pathway, also known as the hyperdirect pathway, and the multisynaptic indirect pathway through the striatum and globus pallidus (GP). In response to motor cortex stimulation (MCS), STN neurons show a distinctive pattern of increased and decreased spike activity. The periods of increased spike activity are related to the excitation of the glutamatergic monosynaptic cortico-subthalamic pathway (N1, Figure 1) (Kitai and Deniau, 1981; Fujimoto and Kita, 1993; Maurice et al., 1998; Nambu et al., 2000; Kolomiets et al., 2001; Magill et al., 2004) and disinhibition via the indirect pathway (N2, Figure 1) (Maurice et al., 1998; Nambu et al., 2000; Magill et al., 2004). In between an inhibitory period is present, which is believed to result from the GABAergic GP connections in the STN-GP-STN feedback loop (P1, Figure 1) (Fujimoto and Kita, 1993; Maurice et al., 1998; Nambu et al., 2000; Magill et al., 2004). After the last excitation, a long-latency, long-duration inhibitory period follows (P2, Figure 1). Electrophysiological studies on cortically evoked subthalamic responses have thus far been focused on the temporal response of the unit activity and LFP on multiple locations within the STN (Fujimoto and Kita, 1993; Maurice et al., 1998; Kolomiets et al., 2001; Magill et al., 2004). None of these studies visualized the spatial organization of the evoked response signal at fixed time stamps, such as the times of the incoming synaptic inputs.

In case of the STN, visualization of the spatial organization of synaptic inputs is important to study the segregation of synaptic pathways in the basal ganglia network and functional segregation in the STN. This is clinically relevant as deep brain stimulation of the STN has shown to be an effective treatment for motor symptoms in patients with Parkinson's disease (Krack et al., 2003; Deuschl et al., 2006; Weaver et al., 2009; Janssen et al., 2014). One of the major hurdles of this therapy is the occurrence of cognitive and limbic alterations in some of the treated patients (Temel et al., 2006; Witt et al., 2008). Many different approaches are explored to selectively target the STN motor region. High resolution imaging has been used to visualize the motor region (Brunenberg et al., 2012), oscillations of neuronal activity (Zaidel et al., 2009) and subthalamic neuronal responses to motor cortex stimulation (Janssen et al., 2012). The CSD approach might provide more insight about the STN functional organization and the differences between unit activity and LFP. This tool might be used to further study cortico-subthalamic pathways in neurodegenerative diseases, which in the end could lead to further optimization of neuromodulative therapies in neurodegenerative diseases.

In this study, MCS evoked LFP and unit activities were simultaneously measured in the subthalamic region of the rat in a high spatial resolution three dimensional (3D) grid consisting 4 × 5 × 16 (antero-posterior × medio-lateral × dorso-ventral) measurement points, containing a volume of 200 × 100 × 1600 μm. These measurements allowed us to perform CSD analysis at the times of the incoming synaptic inputs and to visualize the spatial organization of both components in the electrophysiological signals. The aim of this study was to show the strength of the CSD method to gain new insights into the spatial organization of synaptic pathways in brain structures, such as the clinically relevant STN. Furthermore, the aim was to investigate different cortico-subthalamic pathways, i.e., both the monosynaptic (hyperdirect) and multisynaptic (indirect) pathways and the STN-GP-STN feedback loop.

## Methods

### Experimental design

The experiments described in this paper were conducted on male Sprague Dawley rats (IFFA Credo, St Germain Sur l'Arbresle, France), weighing 250–400 g. Experiments were carried out according to the European Economic Community (86-6091 EEC) and the French National Committee (décret 87/848, Ministère de l'Agriculture et de la Forêt) guidelines for the care and use of laboratory animals and were approved by the Ethical Committee of Centre National de la Recherche Scientifique, Région Aquitaine-Limousin. In each rat, measurements were performed in the right hemisphere. The rats were anesthetized with urethane hydrochloride (1.2 g/kg, i.p. injections, Sigma-Aldrich, Saint-Quentin Fallavier, France) and fixed in a stereotactic frame (Horsley Clarke apparatus, Unimécanique, Epinay sur Seine, France). Body temperature was monitored with a rectal probe and maintained at 37°C with a homeothermic warming blanket (model 50-7061, Harvard Apparatus, Les Ulis, France). Burr holes in the skull were made above the stimulation and recording sites. A saline solution was applied on all exposed cortical areas to prevent dehydration. The microelectrode probe with 16 contacts was used to perform the electrophysiological recordings (A1x16–10mm–100–703–A16, Neuronexus, Ann Arbor, USA). Each contact on the probe has a contact area of 703 μm^2^, and 100 μm inter-electrode distance. The probe was lowered into the brain toward the STN using a microdrive (Microcontroler ESP 300, Newport, Evry, France). Stereotactic coordinates in mm relative to Bregma were: AP −3.8, ML ±2.5, DV −8.0 (Paxinos and Watson, 1998).

When the electrode was in place, the stimulation session started. Recordings of both the unit activity at a sample rate of 22321 Hz and the LFPs at a sample rate of 1395 Hz were performed concurrently with cortical stimulation using the AlphaLab SnR system (AlphaOmega, Nazareth, Israel). After a baseline recording of 2 min, 99 stimuli with an amplitude of 300 μA and 99 stimuli with an amplitude of 600 μA were given. The forelimb region of the motor cortex (coordinates in mm relative to the bregma: AP +3.2, ML ±4.0, DV –2.6) was stimulated with 0.3 ms pulse width and 1.1 Hz frequency ipsilateral to the recording site with two concentric bipolar electrodes (Tan et al., 2010). Electrical stimuli were generated with an isolated stimulator (DS3, Digitimer Ltd., Hertfordshire, UK) triggered by the AlphaLab SnR (AlphaOmega, Nazareth, Israel). Stimulation electrode localization was confirmed histologically.

**Figure 2:**
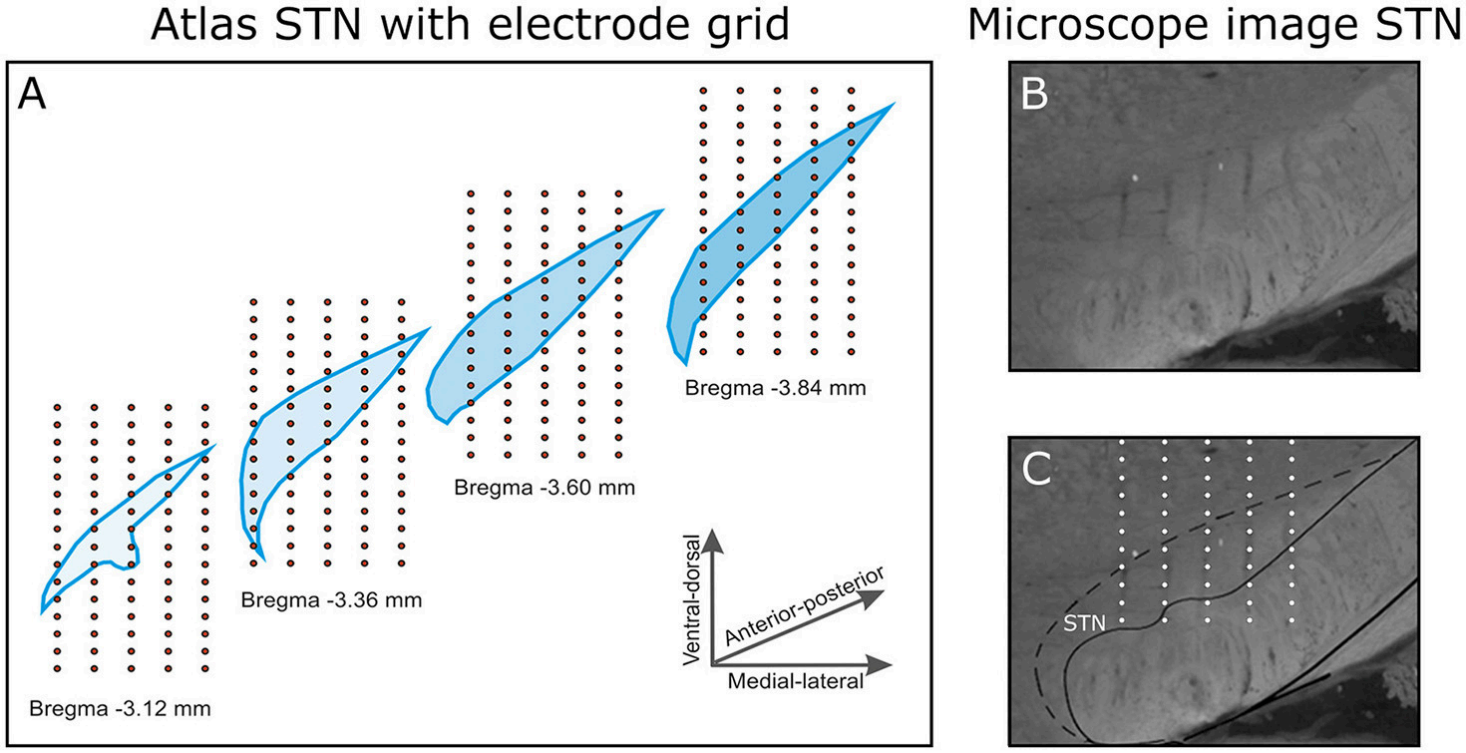
**(A)** The 3D electrode grid inside four coronal STN slices (bregma −3.1 to −3.8 mm in the antero-posterior direction). In total, this gives a 4 × 5 × 16 grid (antero-posterior × medio-lateral × dorso-ventral). Note that the four illustrated slices are 0.24 mm apart, in reality the measurements were performed 0.2 mm apart. **(B)** A selection of a microscope image of a coronal brain slice (Anterior-posterior −3.8 mm relative to Bregma). **(C)** In the brain slice, the electrode trajectories are pointed out within the STN.

After the first stimulation session, the recording probe was retrieved and inserted again at the same depth, but shifted 200 μm in medio-lateral or antero-posterior direction. This was repeated to obtain a total of 20 trajectories, five in medio-lateral direction and four in antero-posterior direction. As the probe consisted of 16 electrode contacts, a 3D measurement grid of 4 × 5 × 16 (antero-posterior × medio-lateral × dorso-ventral) was achieved (Figure 2A. Traces of the electrodes along the trajectories have been verified histologically (Figures 2B,C). Only data from rats in which the traces had been within the STN and in which clear triphasic responses were seen were analyzed, resulting in a total data set from 4 rats. The absolute coordinates of the electrode trajectories with respect to the STN were unfortunately not retrievable.

### Unit activity analysis

The recorded evoked unit activity was visualized by post stimulus time histograms (PSTHs). These histograms were used to visualize the rate and timing of neuronal spike discharges in relation to an external stimulus. PSTHs were generated by using an envelope spike detection method (Dolan et al., 2009). Peaks above the threshold, mean ±3 times the standard deviation (SD), were marked as spikes and principal components analyses were used to classify the waveforms of the detected spikes (Lewicki, 1998). From the classification of the waveforms, the first and second principal components were used for Bayesian clustering, which practices probability density function (Gaussian mixture model) and expectation maximization. Spikes were bin sized at 1 ms. Unit activity was amplitude significant by a threshold of ±3 times the SD based on 100 ms PSTH's of preceding stimulation.

### LFP analysis

First, we checked the channels for high level of noise. If the power between 2 and 200 Hz of the signal during baseline recording exceeded 10 times the average power of all baseline recordings in that particular rat, the measurements at that grid point were rejected. In that case, the average LFP responses of the surrounding grid points were used to interpolate the LFP on the rejected channel. Second, the signals were divided into epochs of 100 ms before stimulation until 500 ms after stimulation. The offset for each epoch was filtered out of the signal using a second order high-pass non-causal Butterworth filter with a cut-off frequency of 1 Hz. All epochs were checked for artifacts; an artifact was detected if the absolute signal in the epoch exceeded 400 μV (note that the signal during the stimulation artifact, from 0 ms to +7 ms relatively to the trigger, was excluded from this criterion). Epochs containing artifacts were rejected. The remaining epochs of the 99 stimuli were averaged per grid point per stimulation type. Subsequently, this average LFP response was smoothed over time and space in dorso-ventral direction, using a third order Savitzky–Golay filter with a window size of 9 samples (Savitzky and Golay, 1964). We only spatially filtered in one axes, because of the lower spatial resolution and smaller range of measurement points in antero-posterior and medio-lateral axes.

### CSD analysis

To estimate the CSD distribution we used the inverse Current Source Density (iCSD) method. The method has been described for one dimensional recordings by Pettersen et al. (Pettersen et al., 2006) and has been generalized to three-dimensional recordings by Leski et al. (2007). The iCSD method is based on linear inversion of the electrostatic forward solution. In the iCSD method, the CSD is assumed to have a certain known distribution class. The distribution class should be parameterized with as many parameters as the number of recorded signals. By using the electrostatic forward solution one can find a linear relation *F* between the CSD distribution and the LFP generated by the CSD on the electrode locations (Equation 1). The linear relation can be used to solve the inverse problem by using the inverse of *F* to calculate the CSD distribution from the recorded LFP signals (Equation 2).

(1) $$\begin{array}{l} \vec{LFP}=F\cdot \vec{CSD} \end{array}$$

(2) $$\begin{array}{l} \vec{CSD}=F^{-1}\cdot \vec{LFP} \end{array}$$

With $\vec{LFP}\;$ the LFP vector ($\vec{LFP}\;in\;R^{\text{320}}$), $\vec{CSD}$ the CSD vector ($\vec{CSD}\;in\;R^{\text{320}}$) and *F* the iCSD transformation matrix. The LFP vector consists of 320 cortically evoked LFPs corresponding to the number of grid points. To describe the CSD distribution we used the natural spline iCSD in which the CSD values within the grid are obtained using natural spline interpolation (Leski et al., 2007). As this approach assumes all sources to be within the measurement grid, an additional boundary condition was introduced. This boundary condition extends the CSD distribution with one layer beyond the original grid, with the grid points in the outer layer having the same value as the nearest CSD value (Leski et al., 2007). Next, the calculated CSD distribution was used to investigate the fast CSD sources and sinks caused by evoked synaptic input from the cortex and GP in the STN. For this, we high-pass filtered the CSD using a second order high-pass non-causal Butterworth filter with a cut-off frequency of 50 Hz. Finally, to determine whether these sources and sinks were of significant amplitude, we used the CSD of 100 ms preceding stimulation and determined the threshold as the mean of this signal ±3 times the SD. When sources and sinks were above or below this threshold, they were considered significant.

### Localization of MCS evoked synaptic activity

For each rat the CSD and PSTH distribution in 3D were evaluated at the points in time at which N1, P1, and N2 occur (Figure 1). At the instant of N1 the excitatory synaptic input will cause an inflow of positively charged ions at the dendrites, which results in negative values in the CSD distribution (sinks). To find the center of the excitatory synaptic pathway in the STN we calculated the center of mass (CoM) of the significant sinks in the evoked CSD distribution at time of N1 (Equation 3–5). At the instant of P1 the inhibitory synaptic input will cause an inflow of negatively charged ions at the dendrites, which results in positive values in the CSD distribution (sources). To find the center of the incoming inhibitory synaptic pathway in the STN we calculated the CoM of the significant sources in the evoked CSD distribution at time of P1 (Equation 3–5).

(3–5) $$\begin{array}{rcl} C_{x} & = & \frac{\sum x.f\left(x,y,z\right)}{\sum f\left(x,yz\right)}\;,\;C_{y}=\frac{\sum y.f\left(x,yz\right)}{\sum f\left(x,y,z\right)}\;,\\ C_{z} & = & \frac{\sum z.f\left(x,y,z\right)}{\sum f\left(x,y,z\right)} \end{array}$$

With C the CoM, *x, y*, and *z* the coordinates within the measurement grid on the, antero-posterior, medio-lateral and dorso-ventral axis respectively, ∑a 3D summation over the measurement grid, and f(*x, y, z*) contains the significant CSD sinks, the significant CSD sources, or the significant PSTH values within the measurement grid.

For each rat we assessed the location of the incoming excitatory and inhibitory synaptic input relatively to the center of the responsive STN cells, i.e., the CoM of the PSTH at time of N1 (Equation 3–5). Also, we assessed the locations of the CoM of the PSTH at N1 and N2, relatively to the center of the excitatory synaptic input at time of N1. A one-way analyses of variance (ANOVA) with *post-hoc* multiple comparison procedure, using a Bonferroni adjustment to compensate for multiple comparisons with a significance level of 0.05, was used to check whether the CoM of the inhibitory and excitatory synaptic input were situated significantly different from each other and from the center of the responsive cells. A paired two tailed *t*-test with a significant level of 0.05 was used to check whether the CoM of the PSTH distribution at time of N1 and N2 were located differently from each other, along the dorso-ventral, antero-posterior, and medio-lateral axis.

## Results

**Figure 3:**
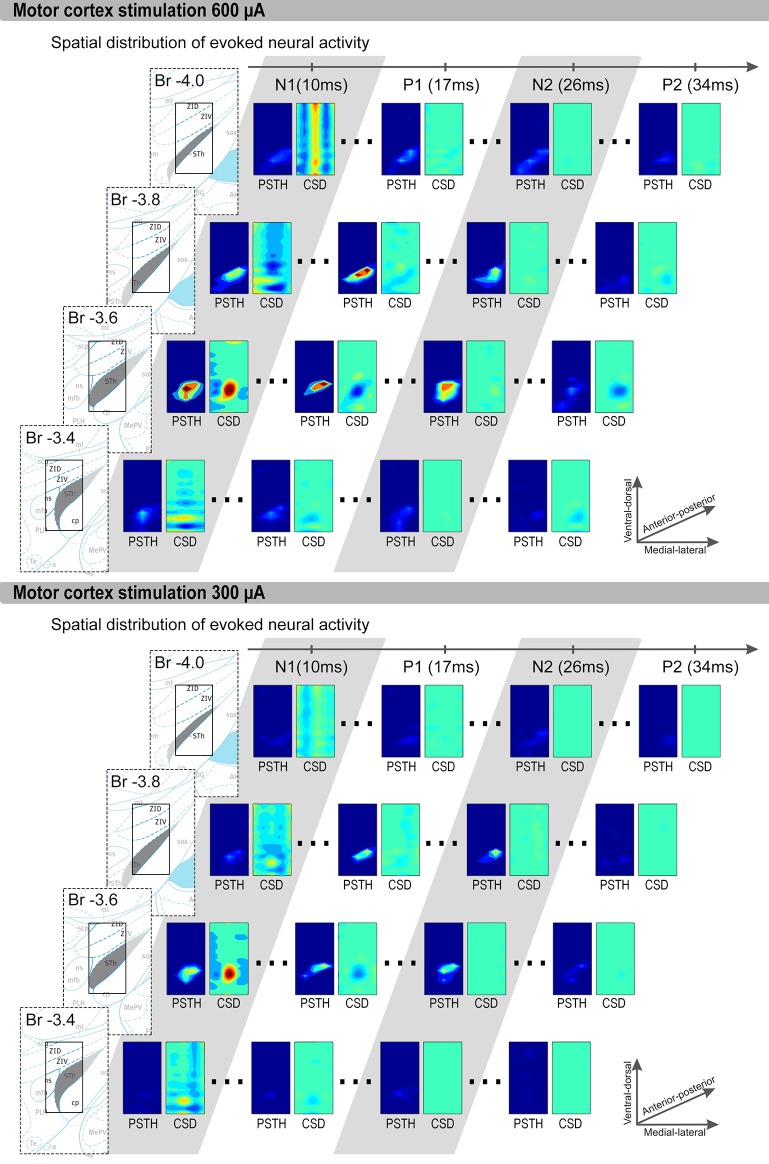
**Representative examples of 600 μA and 300 μA MCS evoked CSD and PSTH distributions are shown**. The four rows contain coronal slices (bregma −3.4 to −4.0 mm). Each row contain, from left to right: First an image of the brain atlas. The atlas is for visualization purposes only, the STN is denoted in gray and the small rectangle is the size of our measurement grid (0.8 mm × 1.5 mm). We used the coronal atlas slices closest to the measurement grid, i.e., bregma −3.36, −3.60, −3.84, and −3.96. Second, the PSTH and CSD distribution at the time of P1, N1, P2, and N2. Only significantly increased spiking rates are shown in the PSTH distributions, and only sinks (red) and sources (blue) with significant strength are shown in the CSD distributions. The x-axis of each rectangle ranges from most medial recording (bregma 2.1 mm) to the most lateral recording (bregma 2.9 mm). The y-axis of each rectangle ranges from most ventral recording to the most dorsal recording.

We first focus on the results obtained during 600 μA MCS (Figure 3). The CSD and unit activity were evaluated for 4 rats at the points in time at which N1, P1, N2, and P2 occur. At the time of N1, the CSD distribution showed a clear local sink corresponding to an excitatory synaptic input, represented by a red area. In the unit activity a locally increased spiking rate can be seen within the grid, also represented by a red area. At the time of P1 the CSD shows a clear local source, represented by a blue area, corresponding to an inhibitory synaptic input near the previously spiking neurons. In the unit activity the spiking rates were reduced. At the time of N2 a locally increased spiking rate can be seen, however no clear sources or sinks were present in the CSD. Finally, at the time of P2 the spiking rate is reduced through the whole area and the CSD showed local source corresponding to an inhibitory synaptic input near the previously spiking neurons. In two rats, during 300 μA MCS, the response was similar as the 600 μA MCS, however the sinks and sources became weaker (Figure 3). The sources at P1 and P2 reduced in strength more dramatically than the sink at N1. In the other two rats the 300 μA MCS did not evoke a similar response.

**Figure 4:**
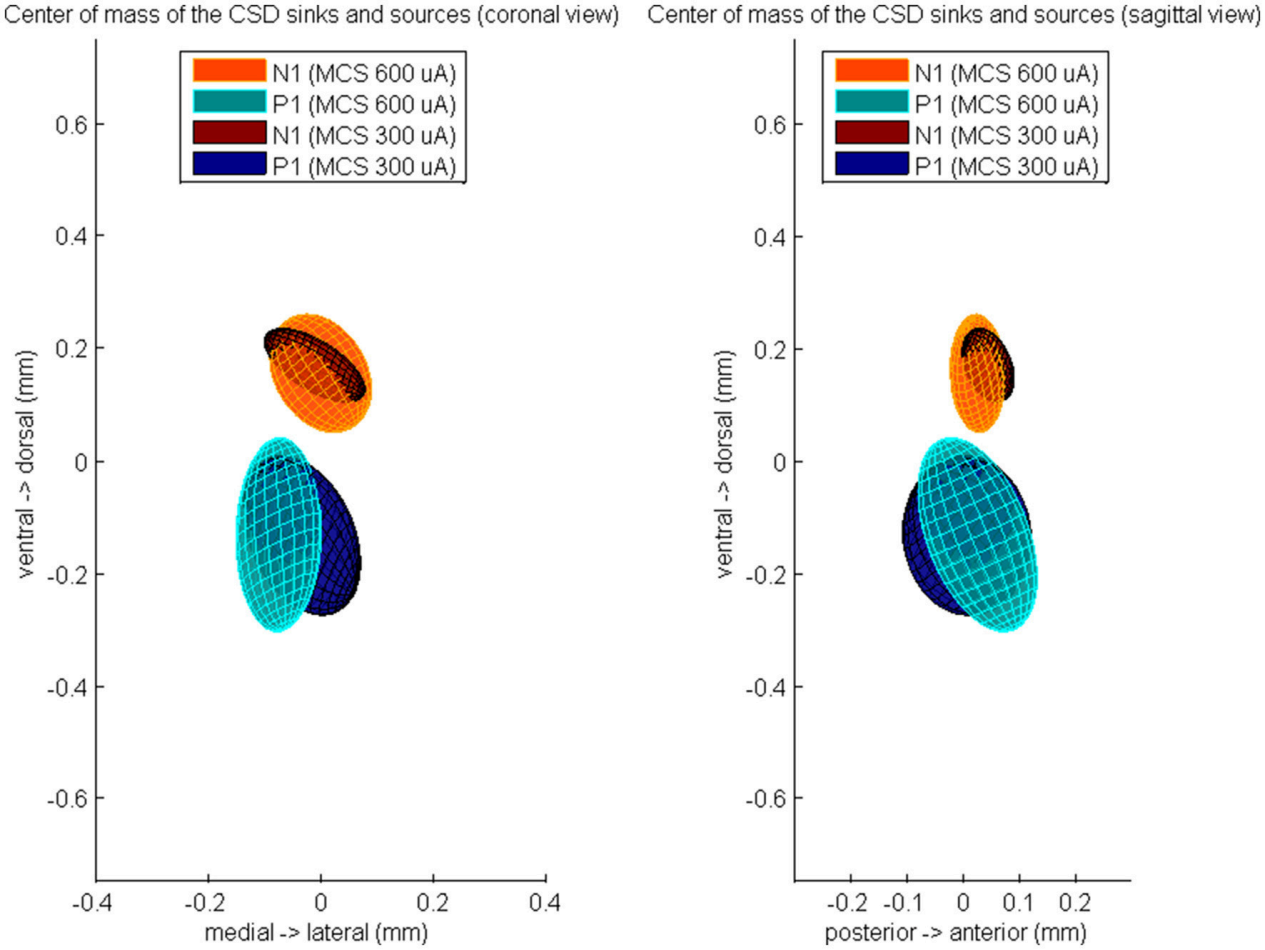
**The averaged center of mass locations of the CSD sources at time of P1 and the CSD sinks at time of N1, relatively to the center of mass of the evoked unit activity at time of N1**. These relative center locations are averaged over all rats which showed a MCS response and is visualized by a color-coded Gaussian ellipsoid. The centroid of the ellipsoid is located on the mean center of mass location and the width of the centroid is the covariance of the center of mass coordinates.

Furthermore, we computed the center of the excitatory and inhibitory synaptic pathways (Figure 4). During 600 μA MCS, the CoM of the CSD sinks at time of N1 was located 26 ± 49 μm anterior, 1 ± 91 μm medial, and 153 ± 105 μm dorsal of the center of the responsive STN cells. The CoM of the CSD sources at time of P1 was located 25 ± 107 μm anterior, 75 ± 76 μm medial, and 134 ± 172 μm ventral of the center of the responsive STN cells. The CoM of the excitatory and inhibitory synaptic inputs were not located significantly different from the center of the responsive cells, however they were located significantly different from each other in the dorsoventral (*p* < 0.01). During 300 μA MCS, the CoM of the CSD sinks at time of N1 was located 44 ± 46 μm anterior, 11 ± 90 μm medial, and 167 ± 66 μm dorsal of the center of the responsive STN cells. The CoM of the CSD sources at time of P1 was located 7 ± 115 μm anterior, 34 ± 104 μm medial, and 135 ± 142 μm ventral of the center of the responsive STN cells. The CoM of the excitatory and inhibitory synaptic inputs were not located significantly different from the center of the responsive cells, however they were located significantly different from each other in the dorsoventral axis (*p* < 0.01).

**Figure 5:**
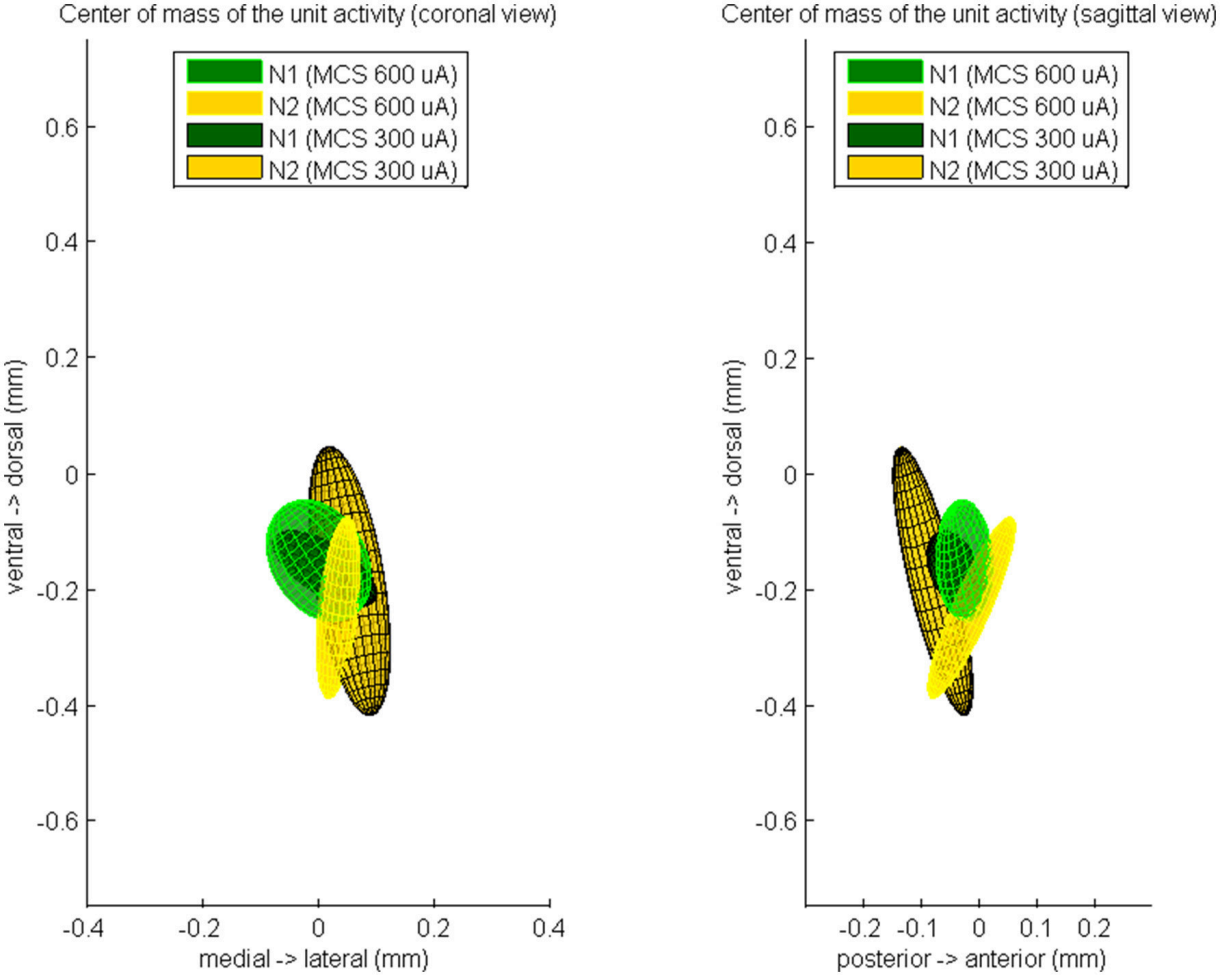
**The averaged center of mass locations of the unit activity at time of N1 and at time of N2, relatively to the center of mass of the evoked CSD source at time of N1**. These relative center locations are averaged over all rats which showed a MCS response and is visualized by a color-coded Gaussian ellipsoid. The centroid of the ellipsoid is located on the mean center of mass location and the width of the centroid is the covariance of the center of mass coordinates.

Finally, we computed the CoM of the unit response during N1 and N2 (Figure 5). During 600 μA MCS, the CoM of the PSTH distribution at time of N1 was located 26 ± 49 μm posterior, 1 ± 91 μm lateral, and 153 ± 105 μm ventral of the CoM of the excitatory synaptic input. The CoM of the unit activity at time of N2 was located 13 ± 76 μm posterior, 34 ± 38 μm lateral, and 235 ± 157 μm ventral of the CoM of the excitatory synaptic input. During 300 μA MCS, the CoM of the PSTH distribution at time of N1 was located 44 ± 46 μm posterior, 11 ± 90 μm lateral, and 167 ± 66 μm ventral of the CoM of the excitatory synaptic input. The CoM of the PSTH distribution at time of N2 was located 80 ± 69 μm posterior, 54 ± 71 μm lateral, and 188 ± 233 μm ventral of the CoM of the excitatory synaptic input. For both stimulation strengths, the CoM of the unit activity at N1 and N2 were not significantly different on any of the three axes.

## Discussion

In this study, LFP and unit activities were simultaneously measured in the subthalamic region with a carefully constructed high resolution measurement grid. For the first time we have performed CSD analysis on MCS evoked LFP responses in the rat STN.

### Interpretation of the MCS evoked response

Previous studies showed that STN neurons display a distinctive temporal pattern of increased and decreased spike activity after cortex stimulation (Fujimoto and Kita, 1993; Maurice et al., 1998; Kolomiets et al., 2001; Magill et al., 2004). The first increased spike activity is due to activation of the glutamatergic monosynaptic cortico-subthalamic pathway. An excitatory synaptic input using glutamate as a neurotransmitter will cause an inflow of positively charged Na^+^ and K^+^ ions at the dendrites (Purves, 2008). Our 3D CSD visualization shows, at the time of this incoming pathway (N1), the inflow of positively charged particles as a strong local current sink within the measurement grid. As expected, the excitatory synaptic input causes an increase of the unit activity within the measurement grid. In addition, the location of the CoM of the excitatory synaptic input was not significantly different than the location of the responsive cells. Intracellular labeling of rat STN neurons shows the dendritic fields are ellipsoidal shaped surrounding the soma (Hammond and Yelnik, 1983), therefore synaptic currents at the dendrites and firing at the soma should be approximately the same location along the different axis.

The inhibitory period which follows is a result of the GABAergic input from GP connections involved in the STN-GP-STN feedback loop (Fujimoto and Kita, 1993; Maurice et al., 1998; Nambu et al., 2000; Magill et al., 2004). An inhibitory GABA synaptic input will cause an inflow of negatively charged Cl- ions at the dendrites. The 3D visualization shows, at the time of this incoming pathway (P1), the inflow of negatively charged particles as a strong local current source within the measurement grid. At the same time, a decrease of spiking activity of the excited STN cells was observed. However, in the example CSD (Figure 3) some of the dorsally located STN cells were not affected by the inhibitory synaptic input. This suggests the local subset of STN cells, which were affected by the excitatory input, triggered the STN-GP-STN feedback loop. However, the evoked inhibition from GP did not cover the complete local subset of STN cells. This finding also corresponds with the computed CoM of sources and sinks in the CSD distribution at the time of N1 and P1.

The CoM of the inhibitory synaptic input from GP was located significantly more ventral than the excitatory input from the cortex. Retrograde and anterograde labeling studies concerning afferent pathways of the rat STN showed that projections from the cerebral cortex were distributed extensively over the STN in only a coarse topographic manner (Afsharpour, 1985; Canteras et al., 1990). In animals with clear topographic organization, such as macaque monkeys, however, the projections from the motor areas appear to be confined to the dorsolateral part of the STN (Monakow et al., 1978). The afferent connections that are involved in the STN-GP-STN feedback loop are suggested to be more precisely organized (Canteras et al., 1990). Anterograde tract-tracing from the GP demonstrated that after deposit of biotinylated dextran amine in the GP, the site of terminal labeling tended to be denser in the ventral border of the STN (Bevan et al., 1997). Another anterograde study using horseradish peroxidase showed that a dense terminal field of GP origin was located in the ventral part of the STN, although it gradually moved more dorsally at the caudal level (Canteras et al., 1990).

After the inhibitory period the spike-rate goes back up at time of N2, due to disinhibition of STN through inhibition of GP by the indirect pathway (Magill et al., 2004). This corresponds to our results. The 3D PSTH distribution showed a local increase of the spike rate. The CSD distribution did not show sources and sinks, meaning that the increase in spike rate is a result of disinhibition and not a result of synaptic input.

The source of the long-latency, long-duration inhibitory period, P2, is thought to result from cortical depression. In our example it looks like that P2 is located in the same area as P1. As P1 is caused by an inhibitory input from the GP, this suggests that P2 is also caused by an inhibitory input from the GP. Also, when comparing the results obtained with 300 μA to those obtained with 600 μA MCS, it is evident that both the area and the amplitude of the source of P2 are reduced more drastically than the sink of N1. This could be explained by the fact that P2 occurred after multiple synaptic stations and thus required temporal-spatial summation to be effective. In contrast, N1 is caused by the monosynaptic cortico-subthalamic pathway.

### The iCSD method

To perform the iCSD method, the responses in the 3D grid have to be measured simultaneously. Our measurements were performed in multiple trials, but by averaging the responses of 99 stimuli; by making sure that the stimulation electrode was not moved during the experiments, we assumed that the responses were very similar to what would have been measured if all the points in the 3D grid were measured simultaneously. The same assumption was made by Leski et al. (2007) to test the iCSD method and they were able to estimate plausible CSD distributions from evoked LFPs. Unfortunately, it is possible that tissue will get damaged due to consecutively inserting the measurement electrode in a small nucleus such as the STN. Remarkably, we kept measuring good quality multiphasic unit activity responses after many consecutive insertions of the measurement electrode.

There are other problems, which complicate the reconstruction of the CSD. The method assumes homogenous isotropic volume conduction of the tissue. The STN is surrounded by myelinated axon bundles (Hamani et al., 2004), which have different conductivities and are highly anisotropic (Nicholson, 1965; Andreuccetti et al., 1997; Haueisen et al., 2002). Besides, as shown in the microscope image of the STN (Figure 2B) the trajectories of the lead can be seen in the tissue. These trajectories also introduce heterogeneous volume conduction in the tissue when they fill up with cerebral fluid. The cerebral fluid has a high conductivity relative to brain tissue (Andreuccetti et al., 1997), the opposite goes for the lead carrying the 16 measurement contacts. The low conductivity of the lead will shield off one side of the measurement electrode.

Finally, in the rats without LFP response, misplacement of either the stimulation or the recording electrode could have occurred. The placing of the recording electrode was checked and confirmed, so misplacement of the recording electrode was not the case. Wearing and tearing of the recording electrode due to blood clotting and cleaning probably affected the impedance of the electrode, which resulted in a decreased signal to noise ratio and also variation in the signal acquisition per measurement point. The iCSD method assumes the exact same signal acquisition for each of the 320 measurement points. When this is not the case, it will result in overfitting of the CSD distribution on the measured signals. Therefore, we included several filtering steps, including spatial as well as temporal filtering, to get a smooth LFP distribution before we used the iCSD method to estimate the CSD distribution.

The iCSD method enables compensation of signal acquisition errors and false assumptions on tissue and electrode impedances, when you know its effect on the measured LFP, by including it in the construction of matrix *F* (Equation 1). Also, instead of using the iCSD method it is possible to use more advanced CSD methods such as the kernel CSD method (Potworowski et al., 2012). This method is based on reproducing kernel Hilbert space and includes cross-validation and ridge regression that address the problem of noise in the data. This method is harder to interpret than a linear method and you have to make new assumption on the size of the sources and sinks.

Despite the problems we addressed, we believe that with a reasonable set of recordings at different sites the CSD reconstruction may provide the basic understanding of different incoming synaptic pathways in a brain structure such as the STN. In contrast to conventional retrograde and anterograde labeling methods, the CSD method allows us to perform *in vivo* experiments without sacrifice of the animal to study synaptic pathways.

### Validity of the results

It should be noted that this study with 4 rats is a proof of principal of the CSD method. The aim of this study was to show the strength of the visualization methods to investigate the spatial organization of both components in the electrophysiological signals. For this, we included only rats with LFPs and unit activity responses similar to those described in earlier reports (Magill et al., 2004). This approach allowed us to use the knowledge from the well described temporal behavior of the evoked response to explain the evoked spatial distributions of the evoked CSD. Our results are well in line with these previous studies, however to gain new insights into the synaptic pathways to the STN and topology of the STN cells, more electrophysiological data needs to be acquired in future studies.

### Future clinical prospects

MCS and simultaneous measurements of the subthalamic response has been performed in PD patients in order to locate the motor area of the STN (Janssen et al., 2012; Zwartjes et al., 2013). The method presented in this paper was able to distinguish the different sources and sinks of the neuronal input in the STN. Novel DBS electrode design are presented, which is capable of high resolution stimulation and recording in different directions (Martens et al., 2011; Bour et al., 2015; van Dijk et al., 2015). In the future this new electrode design enables the CSD approach. The sources and sinks resulting from the CSD approach could be used to optimize the target location for the DBS electrode. In that regard, it has to be determined which location relative to these sources and sinks provides the optimal clinical benefit for the patient. In future, this new approach enables a more precise localization of the STN motor area and could improve surgical outcomes of DBS for PD.

## Conclusion

In this study, we used CSD analysis in the rat to determine the sources and sinks of neuronal input in the STN after cortical stimulation. The CSD method resulted in clear and distinguishable localization of sources and sinks of the neuronal input activity in the STN after MCS. Finally, we showed that the center of the synaptic input of the STN from the MC is located dorsal to the input from GP.

## Author contributions

KV: Writing manuscript, data analyses. MJ: Writing manuscript, measurements. DZ: Writing manuscript, data analyses. YT: Writing project proposal, financing, advising/commenting on manuscript, contributed to the experiment/study design. VV: Writing project proposal, financing, advising/commenting on manuscript, contributed to the experiment/study design. PV: Writing project proposal, financing, advising/commenting on manuscript, contributed to the data analyses and study design. AB: Advising/commenting on manuscript, contributed to the experiment/study design, hosting the animal lab, and measurement equipment. TH: Writing project proposal, financing, advising/commenting on manuscript, contributed to the data analyses and study design.

### Conflict of interest statement

The authors declare that the research was conducted in the absence of any commercial or financial relationships that could be construed as a potential conflict of interest.

## References

[B1] Afsharpour S. (1985). Topographical projections of the cerebral cortex to the subthalamic nucleus. J. Comp. Neurol. 236, 14–28. 10.1002/cne.902360103 2414329

[B2] Andreuccetti D. Fossi R. Petrucci C. (1997). An Internet Resource for the Calculation of the Dielectric Properties of Body Tissues in the Frequency Range 10 Hz - 100 GHz. Florence: IFAC-CNR, Based on data published by C.Gabriel et al. in 1996. Available online at: http://niremf.ifac.cnr.it/tissprop

[B3] Bevan M. D. Clarke N. P. Bolam J. P. (1997). Synaptic integration of functionally diverse pallidal information in the entopeduncular nucleus and subthalamic nucleus in the rat. J. Neurosci. 17, 308–324. 8987757 10.1523/JNEUROSCI.17-01-00308.1997 PMC6793683

[B4] Bour L. J. Lourens M. A. Verhagen R. de Bie R. M. van den Munckhof P. Schuurman P. R.. (2015). Directional recording of subthalamic spectral power densities in parkinson's disease and the effect of steering deep brain stimulation. Brain Stimul. 8, 730–741. 10.1016/j.brs.2015.02.002 25753176

[B5] Brunenberg E. J. Moeskops P. Backes W. H. Pollo C. Cammoun L. Vilanova A.. (2012). Structural and resting state functional connectivity of the subthalamic nucleus: identification of motor STN parts and the hyperdirect pathway. PLoS ONE 7:e39061. 10.1371/journal.pone.0039061 22768059 PMC3387169

[B6] Buzsáki G. (2004). Large-scale recording of neuronal ensembles. Nat. Neurosci. 7, 446–451. 10.1038/nn1233 15114356

[B7] Buzsáki G. Anastassiou C. A. Koch C. (2012). The origin of extracellular fields and currents — EEG, ECoG, LFP and spike*s*. Nat. Rev. Neurosci. 13, 407–420. 10.1038/nrn3241 22595786 PMC4907333

[B8] Canteras N. S. Shammah-Lagnado S. J. Silva B. A. Ricardo J. A. (1990). Afferent connections of the subthalamic nucleus: a combined retrograde and anterograde horseradish peroxidase study in the rat. Brain Res. 513, 43–59. 10.1016/0006-8993(90)91087-W 2350684

[B9] Deuschl G. Schade-Brittinger C. Krack P. Volkmann J. Schäfer H. Bötzel K.. (2006). A randomized trial of deep-brain stimulation for Parkinson's disease. N. Engl. J. Med. 355, 896–908. 10.1056/NEJMoa060281 16943402

[B10] Dolan K. Martens H. C. Schuurman P. R. Bour L. J. (2009). Automatic noise-level detection for extra-cellular micro-electrode recordings. Med. Biol. Eng. Comput. 47, 791–800. 10.1007/s11517-009-0494-4 19468773

[B11] Du J. Blanche T. J. Harrison R. R. Lester H. A. Masmanidis S. C. (2011). Multiplexed, high density electrophysiology with nanofabricated neural probes. PLoS ONE 6:e26204. 10.1371/journal.pone.0026204 22022568 PMC3192171

[B12] Einevoll G. T. Kayser C. Logothetis N. K. Panzeri S. (2013). Modelling and analysis of local field potentials for studying the function of cortical circuits. Nat. Rev. Neurosci. 14, 770–785. 10.1038/nrn3599 24135696

[B13] Freeman J. A. Nicholson C. (1975). Experimental optimization of current source-density technique for anuran cerebellum. J. Neurophysiol. 38, 369–382. 165272 10.1152/jn.1975.38.2.369

[B14] Fujimoto K. Kita H. (1993). Response characteristics of subthalamic neurons to the stimulation of the sensorimotor cortex in the rat. Brain Res. 609, 185–192. 10.1016/0006-8993(93)90872-K 8508302

[B15] Hamani C. Saint-Cyr J. A. Fraser J. Kaplitt M. Lozano A. M. (2004). The subthalamic nucleus in the context of movement disorders. Brain 127(Pt 1), 4–20. 10.1093/brain/awh029 14607789

[B16] Hammond C. Yelnik J. (1983). Intracellular labelling of rat subthalamic neurones with horseradish peroxidase: computer analysis of dendrites and characterization of axon arborization. Neuroscience 8, 781–790. 10.1016/0306-4522(83)90009-X 6866263

[B17] Haueisen J. Tuch D. S. Ramon C. Schimpf P. H. Wedeen V. J. George J. S.. (2002). The influence of brain tissue anisotropy on human EEG and MEG. Neuroimage 15, 159–166. 10.1006/nimg.2001.0962 11771984

[B18] Hubbard J. I. Llinás R. R. Quastel D. M. (1969). Electrophysiological Analysis of Synaptic Transmission. Baltimore, MD: Williams & Wilkins Company.

[B19] Janssen M. L. Duits A. A. Turaihi A. M. Ackermans L. Leentjens A. F. van Kranen-Mastenbroek V.. (2014). Subthalamic nucleus high-frequency stimulation for advanced Parkinson's disease: motor and neuropsychological outcome after 10 years. Stereotact. Funct. Neurosurg. 92, 381–387. 10.1159/000366066 25359232

[B20] Janssen M. L. F. Zwartjes D. G. M. Temel Y. Van Kranen-Mastenbroek V. Duits A. Bour L. J.. (2012). Subthalamic neuronal responses to cortical stimulation. Mov. Disord. 27, 435–438. 10.1002/mds.24053 22213381

[B21] Kipke D. R. Shain W. Buzsáki G. Fetz E. Henderson J. M. Hetke J. F.. (2008). Advanced neurotechnologies for chronic neural interfaces: new horizons and clinical opportunities. J. Neurosci. 28, 11830–11838. 10.1523/JNEUROSCI.3879-08.2008 19005048 PMC3844837

[B22] Kitai S. T. Deniau J. M. (1981). Cortical inputs to the subthalamus: intracellular analysis. Brain Res. 214, 411–415. 10.1016/0006-8993(81)91204-X 7237177

[B23] Kolomiets B. P. Deniau J. M. Mailly P. Menetrey A. Glowinski J. Thierry A. (2001). Segregation and Convergence of Information Flow through the Cortico-Subthalamic Pathways J. Neurosci. 21, 5764–5772. 10.1523/JNEUROSCI.21-15-05764.2001 PMC6762642 11466448

[B24] Krack P. Batir A. Van Blercom N. Chabardes S. Fraix V. Ardouin C.. (2003). Five-year follow-up of bilateral stimulation of the subthalamic nucleus in advanced Parkinson's disease. N. Engl. J. Med. 349, 1925–1934. 10.1056/NEJMoa035275 14614167

[B25] Leski S. Wójcik D. K. Tereszczuk J. Swiejkowski D. A. Kublik E. Wróbel A. (2007). Inverse current-source density method in 3D: reconstruction fidelity, boundary effects, and influence of distant sources. Neuroinformatics 5, 207–222. 10.1007/s12021-007-9000-z 18040890

[B26] Lewicki M. S. (1998). A review of methods for spike sorting: the detection and classification of neural action potentials. Network 9, R53–R78. 10.1088/0954-898X_9_4_001 10221571

[B27] Magill P. J. Sharott A. Bevan M. D. Brown P. Bolam J. P. (2004). Synchronous unit activity and local field potentials evoked in the subthalamic nucleus by cortical stimulation. J. Neurophysiol. 92, 700–714. 10.1152/jn.00134.2004 15044518

[B28] Martens H. C. Toader E. Decré M. M. Anderson D. J. Vetter R. Kipke D. R.. (2011). Spatial steering of deep brain stimulation volumes using a novel lead design. Clin. Neurophysiol. 122, 558–566. 10.1016/j.clinph.2010.07.026 20729143

[B29] Maurice N. Deniau J. M. Glowinski J. Thierry A. (1998). Relationships between the prefrontal cortex and the basal ganglia in the rat Physiology of the corticosubthalamic circuits. J. Neurosci. 18, 9539–9546. 9801390 10.1523/JNEUROSCI.18-22-09539.1998 PMC6792878

[B30] Mitzdorf U. (1985). Current Source-Density method and application in cat cerebral cortex Investigation of evoked potentials and EEG phenomena. Physiol. Rev. 65, 37–100. 3880898 10.1152/physrev.1985.65.1.37

[B31] Monakow K. H.-V. Akert K. Künzle H. (1978). Projections of the precentral motor cortex and other cortical areas of the frontal lobe to the subthalamic nucleus in the monkey. Exp. Brain Res. 33, 395–403. 10.1007/bf00235561 83239

[B32] Nambu A. Tokuno H. Hamada I. Kita H. Imanishi M. Akazawa T.. (2000). Excitatory cortical inputs to pallidal neurons via the subthalamic nucleus in the monkey. J. Neurophysiol. 84, 289–300. 10899204 10.1152/jn.2000.84.1.289

[B33] Nambu A. Tokuno H. Takada M. (2002). Functional significance of the cortico-subthalamo-pallidal e'hyperdirect' pathway. Neurosci. Res. 43, 111–117. 10.1016/S0168-0102(02)00027-5 12067746

[B34] Nicholson P. W. (1965). Specific impedance of cerebral white matter. Exp. Neurol. 13, 386–401. 10.1016/0014-4886(65)90126-3 5847284

[B35] Parent A. Hazrati L. N. (1995). Functional anatomy of the basal ganglia. II. The place of subthalamic nucleus and external pallidum in basal ganglia circuitry. Brain Res. Brain Res. Rev. 20, 128–154. 10.1016/0165-0173(94)00008-D 7711765

[B36] Paxinos G. Watson C. (1998). The Rat Brain in Stereotaxic Coordinates. New York, NY: Academic Press.

[B37] Pettersen K. H. Devor A. Ulbert I. Dale A. M. Einevoll G. T. (2006). Current-source density estimation based on inversion of electrostatic forward solution: effects of finite extent of neuronal activity and conductivity discontinuities. J. Neurosci. Methods 154, 116–133. 10.1016/j.jneumeth.2005.12.005 16436298

[B38] Potworowski J. Jakuczun W. Łȩski S. Wójcik D. (2012). Kernel current source density method. Neural Comput. 24, 541–575. 10.1162/NECO_a_00236 22091662

[B39] Purves D. (2008). Neuroscience. 4th Edn. Sunderland: Sinauer. xvii, 857, G-16, IC-7, I-29.

[B40] Savitzky A. Golay M. J. (1964). Smoothing and differentiation of data by simplified least squares procedures. Anal. Chem. 36, 1627–1639. 10.1021/ac60214a047

[B41] Tan S. K. H. Vlamings R. Lim L. Sesia T. Janssen M. L. F. Steinbusch W. M.. (2010). Experimental deep brain stimulation in animal models. Neurosurgery. 67, 1073–1080. 10.1227/NEU.0b013e3181ee3580 20881571

[B42] Temel Y. Kessels A. Tan S. Topdag A. Boon P. Visser-Vandewalle V. (2006). Behavioural changes after bilateral subthalamic stimulation in advanced Parkinson disease: a systematic review. Parkinsonism Relat. Disord. 12, 265–272. 10.1016/j.parkreldis.2006.01.004 16621661

[B43] van Dijk K. J. Verhagen R. Chaturvedi A. McIntyre C. C. Bour L. J. Heida C.. (2015). A novel lead design enables selective deep brain stimulation of neural populations in the subthalamic region. J. Neural Eng. 12, 046003. 10.1088/1741-2560/12/4/046003 26020096

[B44] Weaver F. M. Follett K. Stern M. Hur K. Harris C. Marks W. J.. (2009). Bilateral deep brain stimulation vs best medical therapy for patients with advanced Parkinson disease: a randomized controlled trial. JAMA 301, 63–73. 10.1001/jama.2008.929 19126811 PMC2814800

[B45] Witt K. Daniels C. Reiff J. Krack P. Volkmann J. Pinsker M. O. (2008). Neurophysiological and psychiatric changes after deep brain stimulation for Parkinson's disease: a randomised, multicentre study. Lancet Neurol. 7, 605–614. 10.1016/S1474-4422(08)70114-5 18538636

[B46] Zaidel A. Spivak A. Shpigelman L. Bergman H. Israel Z. (2009). Delimiting subterritories of the human subthalamic nucleus by means of microelectrode recordings and a Hidden Markov Model. Mov. Disord. 24, 1785–1793. 10.1002/mds.22674 19533755

[B47] Zwartjes D. G. Janssen M. L. Heida T. Van Kranen-Mastenbroek V. Bour L. J. Temel Y.. (2013). Cortically evoked potentials in the human subthalamic nucleus. Neurosci. Lett. 539, 27–31. 10.1016/j.neulet.2013.01.036 23384566

